# Evaluating artificial intelligence-generated synthesized mammography as a standalone alternative to digital mammography with or without tomosynthesis

**DOI:** 10.1007/s11604-026-02016-3

**Published:** 2026-05-25

**Authors:** Takayoshi Uematsu, Kazuaki Nakashima, Taiyo L. Harada, Sachiko Yuen, Ichiro Isomoto, Hatsuko Nasu, Tatsuya Igarashi, Shoko Abe, Kouichirou Oosawa, Takashi Itoh, Yuji Maruo, Hidetaka Kobayashi, Yoshihiro Sakai

**Affiliations:** 1 Division of Breast Imaging and Breast Interventional Radiology, Shizuoka Cancer Center Hospital, Shizuoka, 411-8777 Japan; 2 Department of Breast Surgery and Oncology, Shinko Hospital, Kobe, Japan; 3 Department of Radiology, St. Francis Hospital, Nagasaki, Japan; 4 Department of Radiology, Hamamatsu University School of Medicine, Shizuoka, Japan; 5 Department of Radiology, Nishiyaidu Health Check-Up Center, Shizuoka, Japan; 6 Department of Radiology, NHO Shizuoka Medical Center, Shizuoka, Japan; 7 Osawa Clinic, Shizuoka, Japan; 8 Division of Surgery, Seirei-Numazu Hospital, Shizuoka, Japan; 9 Onaribashi Sakae Clinic, Shizuoka, Japan; 10 Sakai Clinic, Shizuoka, Japan

**Keywords:** Breast neoplasms, Mammography, Image processing, Computer-assisted, Artificial intelligence, Mass screening

## Abstract

**Purpose:**

To evaluate whether standalone synthesized mammography (SM) can maintain or improve diagnostic accuracy while reducing reading time and radiation dose, compared to digital breast tomosynthesis (DBT) with digital mammography (DM) or DM alone.

**Materials and methods:**

This was a retrospective study. SM images were generated using an AI-supported DBT reading method that synthesizes key features from DBT images without displaying marks or outlines. Twelve readers independently evaluated 200 breasts (including 50 cancers) using SM alone and DM with or without DBT acquired from October 2022 to January 2023. Diagnostic performance was assessed using area under the receiver operating characteristic curve (AUC), sensitivity, specificity, reading time, and radiation dose. A multireader, multicase statistical model was applied. Sample size and power calculations were based on estimates from a prior study.

**Results:**

SM alone achieved an AUC of 0.912, which did not differ significantly from that of DBT with DM (0.906; p = 0.538) or DM alone (0.897; p = 0.13). Sensitivity improved from 88.1% (DBT with DM) and 86.6% (DM) to 90.9% with SM, with a significant improvement over DM (p = 0.042). Specificity remained comparable (SM: 84.5%; DBT with DM: 85.9%; DM: 86.5%). SM alone reduced mean reading time by 53.1% (122.6 to 57.5 s; p < 0.001) and radiation dose by 39% (1.73 vs. 2.84 mGy) compared with DBT with DM.

**Conclusions:**

Standalone AI-generated SM preserved the diagnostic accuracy of DBT with DM while reducing reading time and radiation dose, and improved sensitivity over DM without loss of specificity.

## Introduction

Digital breast tomosynthesis (DBT), a pseudo–three-dimensional extension of digital mammography (DM) reconstructing 1-mm slices from low-dose projections [1], has been widely adopted in breast cancer screening [2, 3]. DBT with DM improves cancer detection and reduces false positives compared with DM alone [4], and is associated with lower interval cancer rates [5], although concerns regarding overdiagnosis remain.

However, widespread adoption is limited by increased radiation exposure and longer reading time [1]. Synthesized mammography (SM) addresses radiation concerns by reconstructing 2D images from DBT without additional exposure, while maintaining diagnostic performance [6]. DBT also increases interpretation time, burdening workflow amid radiologist shortages and routine double reading [7, 8]. The Malmö trial demonstrated that single-view MLO DBT may improve efficiency while reducing dose [9].

Artificial intelligence (AI) may mitigate these limitations by reducing reading time while preserving accuracy. Conventional AI-assisted DBT systems support radiologists through automated, CAD-like second-opinion diagnostic assistance and have been shown to reduce recall rates and workload by approximately 20% [10]. More broadly, AI may enhance medical imaging by improving image quality and enabling detection of subtle features that may be overlooked by human readers, thereby supporting diagnostic accuracy. Uematsu et al. developed an AI-generated SM that extracts key features from DBT without visual markers, enabling efficient interpretation and performance comparable to DBT with DM at lower dose and reading time [11, 12]. Recent deep learning approaches may further enhance efficiency [13]. If standalone SM can replace DBT with DM in screening, it may streamline workflow and reduce radiologist workload amid workforce shortages. Therefore, this study aimed to evaluate whether AI-generated SM can serve as a practical standalone alternative to DBT with DM in a screening-oriented interpretation task, with a particular focus on its potential to maintain diagnostic performance while improving workflow efficiency and reducing radiation exposure.

## Materials and methods

### Study overview and data acquisition

This multireader, multicase study was approved by the institutional review board, and written informed consent was obtained from all participants. FUJIFILM provided the imaging equipment and technical support; however, the reference radiologist (T.U.), who established the ground truth, was independent of FUJIFILM and retained full control over the data and publication decisions. A total of 193 DBT + DM cases (bilateral two-view) were acquired at Shizuoka Cancer Hospital between October 2022 and January 2023 using a DBT system (AMULET SOPHINITY; FUJIFILM; ± 7.5° sweep, 19 projections, 9 s per view). Radiation dose was automatically calculated from exposure parameters and extracted from DICOM tags. Reader performance was assessed on a per-breast basis using MLO images only to account for reading time and attention span [11, 12]. This approach is consistent with the Japanese national breast cancer screening program, which recommends single-view MLO mammography every two years for women aged ≥ 50 years, while two-view screening is recommended for women in their 40 s [14].

Study design and sample size calculation

This multireader, multicase study compared SM alone (AI-supported DBT) with DM ± DBT. Twelve readers evaluated 100 cases (200 breasts: 50 malignant and 12 biopsy-proven benign). Each case was interpreted twice—once with DBT + DM and once with SM alone—with a minimum 4-week washout period to minimize recall bias. Sample size estimation, based on a prior study [11] assuming a mean AUC difference of 0.023, indicated that 50 malignant breasts, 150 normal or benign breasts, and 12 readers would provide 81.3% power (α = 0.05) using the method of Hills et al. [15].

### Dataset

**Fig. 1:**
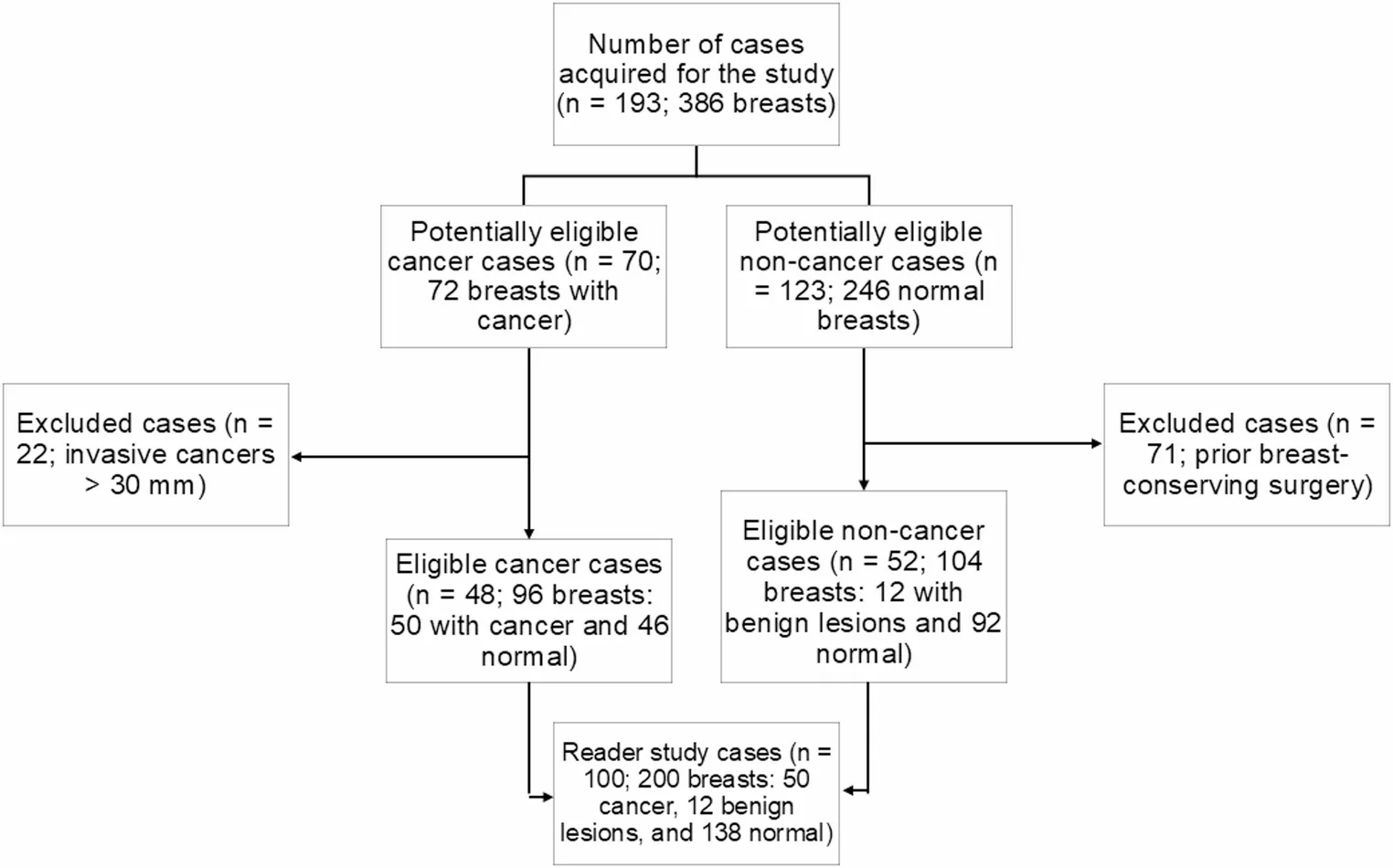
Data set selection flowchart. The two breast cancer cases had bilateral breast disease. All breast cancer lesions were histologically confirmed and detected using ultrasound and/or magnetic resonance imaging. Exclusions were prior breast-conserving surgery (n = 71) and invasive cancers larger than 30 mm (n = 22) to reduce recall bias and exclude the less challenging diagnoses

The study included 193 consecutive cases that presented to or were referred to our institution between October 2022 and January 2023 for diagnostic evaluation following abnormal or equivocal screening findings, clinical symptoms, or postoperative follow-up after breast-conserving surgery. Of these, 100 cases (200 breasts: 50 malignant, 12 biopsy-proven benign, and 138 normal confirmed by 2-year follow-up) met the eligibility criteria (Fig. 1). The exclusion criteria were prior breast-conserving surgery (n = 71) and invasive cancers larger than 30 mm (maximum tumor diameter based on imaging findings), which could be accompanied by clinical symptoms, to minimize recall bias and exclude less challenging cases. The final cohort ranged in age from 32 to 87 years (median, 56 years), which reflects the epidemiologic pattern in Japanese women, in whom breast cancer incidence rises from the late 30 s and increases sharply through the 40 s and beyond, contributing to the disease burden among women aged 30–64 [16]. Lesion types included 30 masses (28 malignant, 2 benign), 3 masses with calcifications (all malignant), 1 mass with distortion (malignant), 4 distortions (2 malignant), and 24 calcification-only cases (16 malignant). Cancer types were 11 ductal carcinoma in situ, 2 microinvasive carcinomas, 33 invasive ductal carcinomas, and 1 each of mucinous carcinoma, encysted papillary carcinoma, apocrine carcinoma, and invasive lobular carcinoma. Lesion sizes ranged from 6 to 58 mm (median, 16 mm). Lesion size was determined from surgical pathology findings when available, or from the longest linear dimension measured on study images.

### Generation of new standalone SM

A pre-release version of FUJIFILM’s image processing system was used to generate the new SM. The system synthesizes a 2D mammogram from DBT slices through motion correction and AI-based detection of characteristic breast structures, followed by generation of a representative 2D image. This updated processing framework incorporates structure-specific feature detection and refinement of image synthesis, which may enhance lesion conspicuity and facilitate efficient interpretation compared with earlier implementations. Spherical and radial structures are detected using deep learning with a 3D U-Net convolutional neural network [17], while microcalcification-like structures are identified using filter-based structural recognition.

### Readers

All 12 readers were certified by the Japan Central Organization on Quality Assurance of Breast Cancer Screening. Reader experience ranged from 7 to 31 years (median, 25 years). Five readers with > 2500 DBT cases in the prior 5 years were classified as experienced, and the remaining seven with minimal or no DBT experience as novice. All readers completed training with 25 DBT + DM and SM cases (not included) to familiarize themselves with the workstation and protocol.

### Reading methods

Readers were blinded to case distribution, lesion characteristics, clinical information, and prior imaging. Each case was interpreted twice in two sessions: (a) DM followed by DBT with DM, and (b) SM alone, with a ≥ 4-week washout period to minimize recall bias. In session (a), DM was read before DBT. Modality order was fixed, whereas case order was randomized for each reader in each session. Lesions were scored on a five-point scale (1 = normal to 5 = malignant) based on the Japanese breast cancer screening classification system, which is widely used in clinical practice in Japan. In this system, categories 1 and 2 indicate negative or benign findings not requiring further evaluation, whereas categories 3–5 correspond to findings requiring additional diagnostic assessment. Accordingly, probability of malignancy (0–100%) was recorded only for cases scored as category ≥ 3, reflecting clinically actionable findings in a screening context, and the most suspicious lesion was annotated. Reading time per case was automatically recorded by the workstation from image display to completion of data entry.

### Statistical analysis

Inter-reader agreement for binary classifications (score ≥ 3) was assessed using Fleiss’ kappa with 95% confidence intervals for each modality (SM, DBT with DM, and DM alone) on a per-breast basis. Confidence intervals were estimated using a normal approximation, and agreement strength was categorized from none to almost perfect. Diagnostic accuracy was evaluated using mean AUCs with a random-reader, random-case model to account for within-patient and between-reader correlations. Bootstrap percentile confidence intervals and P values were obtained from 100,000 bootstrap samples. Reading times were compared using a linear mixed model with subject and reader as random effects. All tests used a two-sided significance level of 0.05 and were performed using R (v4.0.2; R Foundation for Statistical Computing).

## Results

### Radiation dose associated with each examination

The mean compressed breast thickness during DBT with DM was 38.1 ± 12.7 mm. The mean glandular dose per view was 1.11 ± 0.26 mGy for DM and 1.73 ± 0.49 mGy for DBT. Use of the new SM reduced the average dose by 39% compared with DBT with DM (1.73 mGy vs. 2.84 mGy).

### Reader performance

Inter-reader agreement (score ≥ 3) among the 12 readers was substantial for all three modalities, with kappa values of 0.634 (95% CI, 0.616–0.651) for SM, 0.638 (95% CI, 0.620–0.655) for DBT with DM, and 0.675 (95% CI, 0.658–0.692) for DM.

**Fig. 2:**
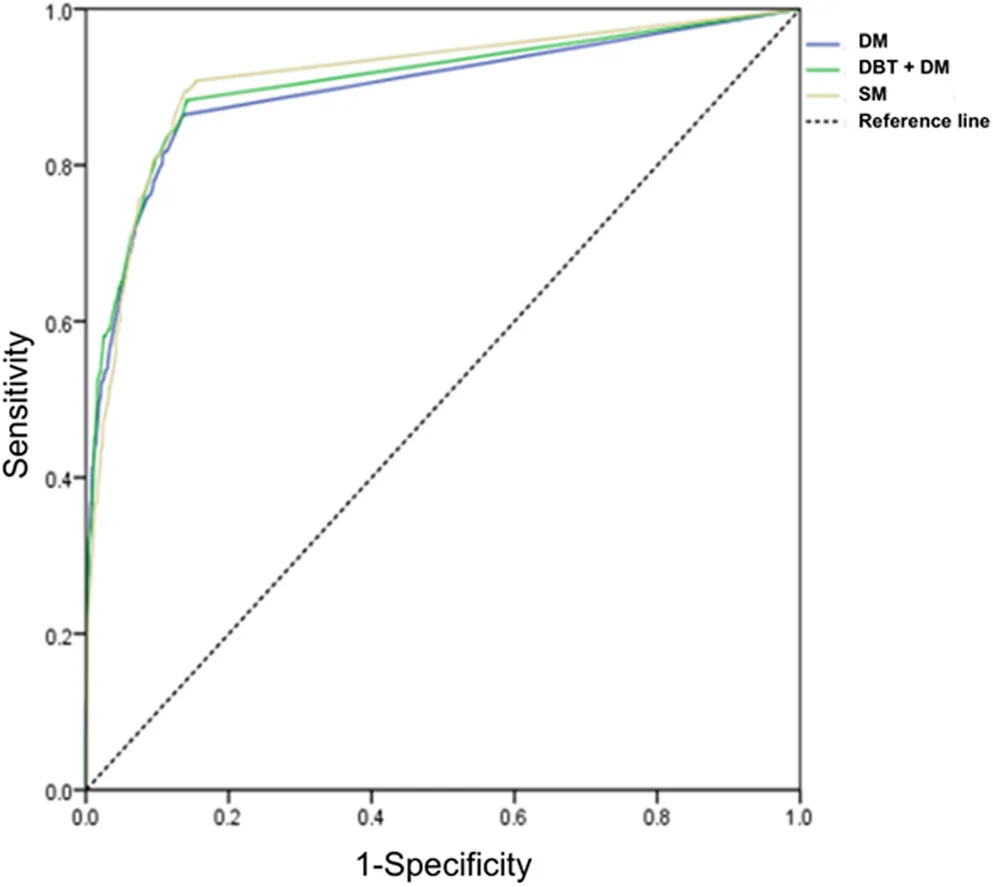
Overall receiver operating characteristic curve based on probability-of-malignancy ratings for individual breasts

The mean AUC values were 0.912 for SM, 0.906 for DBT with DM, and 0.897 for DM (Table 1, Fig. 2). SM alone did not differ significantly from DBT with DM (95% CI − 0.013 to 0.026; p = 0.538) or from DM (95% CI − 0.005 to 0.037; p = 0.13). Seven of the 12 readers (58%) achieved higher AUC with SM than with DBT with DM, and nine (75%) achieved higher AUC with SM than with DM. Among the five experienced readers, the mean AUC for SM was significantly higher than for DM (95% CI 0.008 to 0.071; p = 0.011). Among the seven novice readers, the mean AUCs were 0.906 for SM, 0.911 for DBT with DM, and 0.904 for DM.

**Table 1:** Diagnostic accuracy (AUC) by reader experience and imaging modality

Reader group	Reader ID	SM (AUC, 95% CI)	DBT + DM (AUC, 95% CI)	DM (AUC, 95% CI)	SM vs. DBT + DM ΔAUC	p value	SM vs. DM ΔAUC	p value	DM vs. DBT + DM ΔAUC	p value
All readers (n = 12)	Mean	0.912 (0.892–0.932)	0.906 (0.885–0.926)	0.897 (0.874–0.918)	− 0.006 (− 0.013–0.026)	0.538	0.015 (− 0.005–0.037)	0.130	0.009 (− 0.008–0.028)	0.283
Experienced readers (n = 5)	Mean	0.928 (0.886–0.960)	0.905 (0.862–0.941)	0.888 (0.844–0.927)	0.023 (− 0.005–0.050)	0.114	0.040 (0.008–0.071)	0.011*	0.017 (− 0.008–0.043)	0.198
Novice readers (n = 7)	Mean	0.906 (0.863–0.941)	0.911 (0.868–0.945)	0.904 (0.867–0.935)	− 0.005 (− 0.003–0.017)	0.716	0.002 (− 0.020–0.025)	0.871	− 0.007 (− 0.014–0.027)	0.536

AUC, area under the receiver operating characteristic curve; CI, confidence interval; ΔAUC, difference in AUC; SM, synthesized mammography; DBT, digital breast tomosynthesis; DM, digital mammography

*Statistically significant difference (p < 0.05)

Sensitivity increased from 88.1% with DBT with DM to 90.9% with SM alone (95% CI − 0.0015 to 0.0072; p = 0.152) and increased significantly from 86.6% with DM to 90.9% with SM (95% CI 0.0001 to 0.089; p = 0.042). Specificity was 84.5% for SM, 85.9% for DBT with DM, and 86.5% for DM, with no significant differences (Table 2). Among the five experienced readers, mean sensitivities were 92.5% for SM, 85.7% for DBT with DM, and 83.3% for DM. SM sensitivity was significantly higher than both DBT with DM (95% CI 0.018 to 0.121; p = 0.007) and DM alone (95% CI 0.039 to 0.147; p < 0.0001). Mean specificities were 87.5% for SM, 88.7% for DBT with DM, and 89.8% for DM, with no significant differences. Among the seven novice readers, mean sensitivities were 89.8% for both SM and DBT with DM, and 89.0% for DM, with no significant differences. Mean specificities were 82.3% for SM, 83.9% for DBT with DM, and 84.1% for DM, also without significant differences.

**Table 2:** Sensitivity and specificity by reader experience and imaging modality

Reader group	Reader ID	Parameter	SM (%)	DBT + DM (%)	DM (%)	SM vs. DBT + DM Δ (95% CI)	p value	SM vs. DM Δ (95% CI)	p value	DM vs. DBT + DM Δ (95% CI)	p value
All readers (n = 12)	Mean	Sensitivity	90.9	88.1	86.6	0.057 (− 0.015–0.072)	0.152	0.047 (0.0001–0.089)	0.042	0.029 (− 0.026–0.055)	0.414
Experienced readers (n = 5)	Mean	Sensitivity	92.5	85.7	83.3	0.063 (0.018–0.121)	0.007	0.086 (0.039–0.147)	< 0.001	0.024 (− 0.023–0.071)	0.254
Novice readers (n = 7)	Mean	Sensitivity	89.8	89.8	89.0	0.002 (− 0.049–0.047)	0.891	0.009 (− 0.041–0.059)	0.628	0.008 (− 0.039–0.057)	0.607
All readers (n = 12)	Mean	Specificity	84.5	85.9	86.5	− 0.014 (− 0.046–0.016)	0.387	− 0.020 (− 0.052–0.012)	0.229	− 0.006 (− 0.032–0.020)	0.720
Experienced readers (n = 5)	Mean	Specificity	87.5	88.7	89.8	− 0.012 (− 0.041–0.018)	0.470	− 0.028 (− 0.053–0.008)	0.161	− 0.021 (− 0.035–0.015)	0.470
Novice readers (n = 7)	Mean	Specificity	82.3	83.9	84.1	− 0.016 (− 0.056–0.023)	0.465	− 0.018 (− 0.059–0.022)	0.409	− 0.002 (− 0.035–0.031)	0.970

CI, confidence interval; Δ, difference between modalities; SM, synthesized mammography; DBT, digital breast tomosynthesis; DM, digital mammography

*Statistically significant difference (p < 0.05)

### Sensitivity and specificity by lesion type and breast density

These subgroup analyses were exploratory and based on limited sample sizes; therefore, the findings should be interpreted cautiously. Specificity could not be assessed in certain subgroups because of the small number of benign cases.

Calcification-only lesions: SM showed the highest mean sensitivity (91.4%) compared with DBT with DM (89.8%) and DM alone (90.3%) (Table 3). Specificity was not assessed due to the small number of benign calcification-only lesions (n = 8). Among experienced readers, SM sensitivity exceeded DBT with DM (92.9% vs. 87.8%; 95% CI 0.002 to 0.120; p < 0.0001).

**Table 3:** Sensitivity and specificity by lesion type and breast density, stratified by reader DBT experience

Reader group	Parameter	SM (%)	DBT + DM (%)	DM (%)	SM vs. DBT + DM (95% CI)	p	SM vs. DM (95% CI)	p	DM vs. DBT + DM (95% CI)	p
*A. Calcification-only lesions*										
All readers (n = 12)	Sensitivity	91.4	89.8	90.3	− 0.042–0.078	0.417	− 0.061–0.082	0.603	− 0.070–0.061	0.930
Experienced (n = 5)	Sensitivity	92.9	87.8	87.8	0.002–0.120	< 0.001	− 0.038–0.145	0.163	− 0.078–0.082	0.772
Novice (n = 7)	Sensitivity	90.4	91.3	92.2	− 0.082–0.063	0.945	− 0.094–0.059	0.853	− 0.082–0.061	0.956
*B. Lesions other than calcification-only*										
All readers (n = 12)	Sensitivity	90.7	87.3	84.9	− 0.022–0.092	0.187	0.007–0.117	0.022	− 0.029–0.076	0.280
Experienced (n = 5)	Sensitivity	92.5	84.8	81.2	0.010–0.150	0.017	0.048–0.182	< 0.001	− 0.029–0.096	0.188
Novice (n = 7)	Sensitivity	89.5	89.2	87.5	− 0.058–0.068	0.787	− 0.040–0.086	0.415	− 0.047–0.078	0.492
*C. Dense breasts*										
All readers (n = 12)	Sensitivity	87.6	84.4	87.0	− 0.027–0.093	0.208	− 0.051–0.064	0.691	− 0.081–0.029	0.449
Experienced (n = 5)	Sensitivity	88.7	81.7	83.1	0.0001–0.141	0.020	− 0.014–0.131	0.099	− 0.072–0.039	0.766
Novice (n = 7)	Sensitivity	87.0	86.5	89.9	− 0.064–0.076	0.724	− 0.090–0.029	0.488	− 0.102–0.029	0.424
All readers (n = 12)	Specificity	83.9	86.6	87.3	− 0.067–0.012	0.187	− 0.077–0.009	0.132	− 0.040–0.027	0.768
Experienced (n = 5)	Specificity	88.2	90.0	92.4	− 0.056–0.019	0.401	− 0.083–0.0001	0.059	− 0.054–0.007	0.156
Novice (n = 7)	Specificity	80.8	84.2	83.8	− 0.085–0.016	0.198	− 0.086–0.023	0.309	− 0.039–0.048	0.770
*D. Non-dense breasts*										
All readers (n = 12)	Sensitivity	94.6	92.1	86.2	− 0.038–0.089	0.341	0.019–0.157	0.007	0.0001–0.125	0.040
Experienced (n = 5)	Sensitivity	97.0	90.1	83.5	− 0.012–0.147	0.052	0.062–0.217	< 0.001	0.0001–0.145	0.049
Novice (n = 7)	Sensitivity	93.1	93.7	88.2	− 0.078–0.063	0.988	− 0.031–0.131	0.175	− 0.014–0.123	0.066
All readers (n = 12)	Specificity	85.3	84.9	85.3	− 0.045–0.052	0.799	− 0.047–0.047	0.926	− 0.045–0.037	0.909
Experienced (n = 5)	Specificity	86.6	86.9	86.3	− 0.049–0.043	0.990	− 0.041–0.047	0.802	− 0.037–0.050	0.691
Novice (n = 7)	Specificity	84.4	83.6	84.6	− 0.053–0.070	0.741	− 0.062–0.056	0.992	− 0.059–0.040	0.755

CI, confidence interval; SM, synthesized mammography; DBT, digital breast tomosynthesis; DM, digital mammography

*Statistically significant difference (p < 0.05)

*Specificity was not evaluated for sections A and B due to the limited number of benign cases

**Fig. 3:**
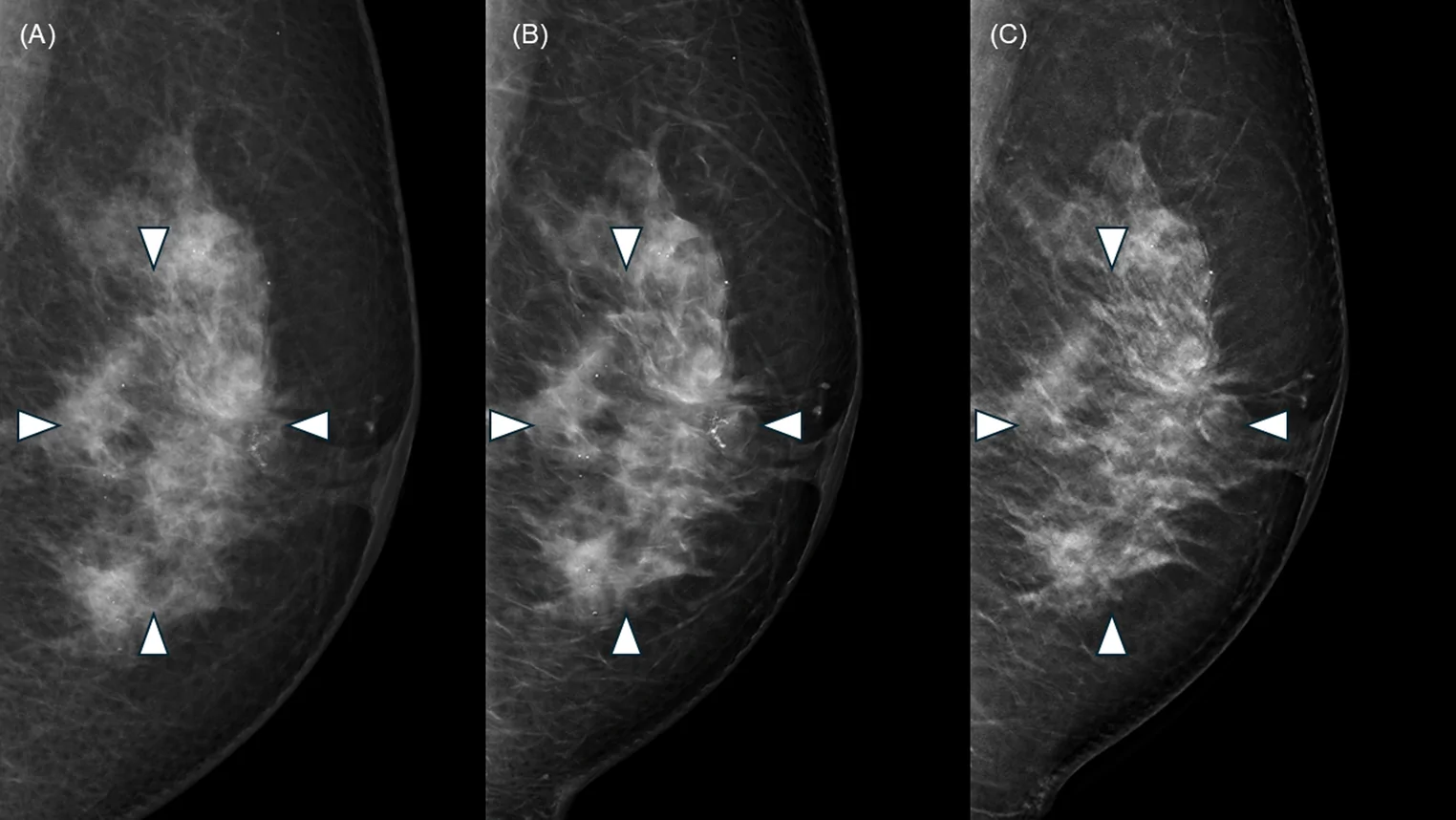
Images from a 46-year-old Japanese woman with invasive lobular carcinoma in a heterogeneously dense left breast. Architectural distortion (arrowheads) is more clearly visualized on the synthesized mammogram (SM) (**B**) than on the digital mammogram (DM) (**A**). The SM image (**B**) is comparable to the corresponding digital breast tomosynthesis (DBT) image (**C**)

**Fig. 4:**
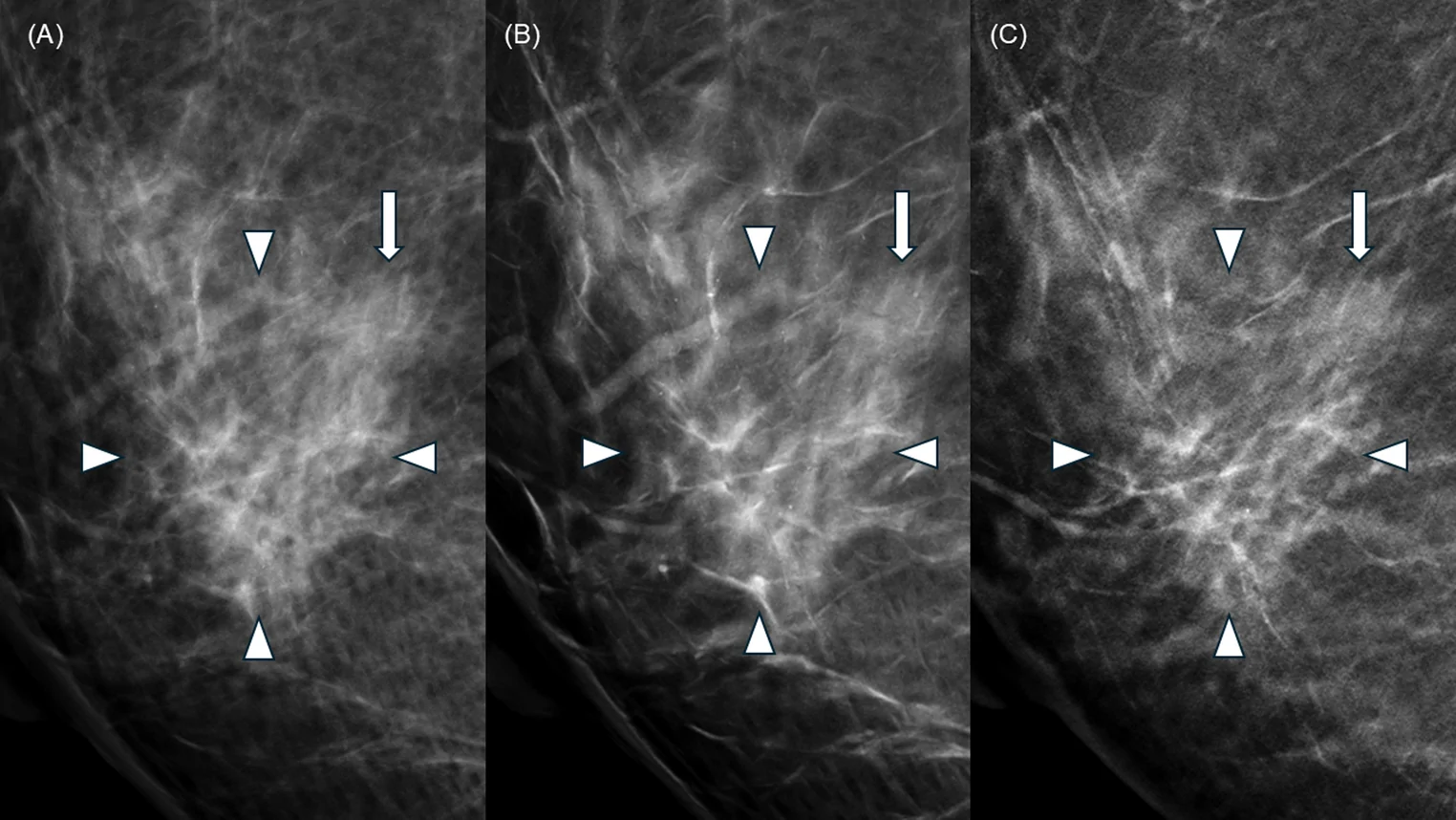
Images from a 60-year-old Japanese woman with apocrine carcinoma and associated ductal carcinoma in situ (DCIS) in a heterogeneously dense right breast. **A** small mass (arrow) with surrounding amorphous calcifications (arrowheads) is more clearly visualized on the synthesized mammogram (SM) (**B**) and the corresponding digital breast tomosynthesis (DBT) image (**C**) than on the digital mammogram (DM) (**A**). Among these, the SM image (**B**) depicts the surrounding amorphous calcifications most distinctly

Lesions other than calcification-only (mass, mass with calcifications, distortion): Mean sensitivities were 90.7% (SM), 87.3% (DBT with DM), and 84.9% (DM), with no significant differences (Table 3, Figs. 3 and 4). Among experienced readers, SM sensitivity was higher than DBT with DM (92.5% vs. 84.8%; 95% CI 0.010 to 0.150; p = 0.017) and DM alone (92.5% vs. 81.2%; 95% CI 0.048 to 0.182; p < 0.0001).

Dense breasts: Mean sensitivities were 87.6% (SM), 84.4% (DBT with DM), and 87.0% (DM), with no significant differences; specificities were 83.9%, 86.6%, and 87.3%, respectively (Table 3). Among experienced readers, SM sensitivity was higher than DBT with DM (88.7% vs. 81.7%; 95% CI 0.0001 to 0.141; p = 0.02).

Non-dense breasts: Mean sensitivities were 94.6% (SM), 92.1% (DBT with DM), and 86.2% (DM) (Table 3). SM sensitivity was higher than DM (94.6% vs. 86.2%; 95% CI 0.019 to 0.157; p = 0.007) and showed a non-significant increase versus DBT with DM (94.6% vs. 92.1%; 95% CI − 0.038 to 0.089; p = 0.341). Specificities were similar across SM (85.3%), DBT with DM (84.9%), and DM (85.3%). Among experienced readers, SM exceeded DM (97.0% vs. 83.5%; 95% CI 0.062 to 0.217; p < 0.0001) and trended higher than DBT with DM (97.0% vs. 90.1%; 95% CI − 0.012 to 0.147; p = 0.052).

### Reading time changes

Mean reading time decreased by 53.1% (95% CI 58.1–49.0%; p < 0.001), from 122.6 s with DBT + DM to 57.5 s with SM alone. Mean reading times for SM and DM were comparable (57.4 s vs. 55.4 s). This reduction was observed consistently in both experienced and novice readers (Table 4). The median reading times, with interquartile ranges shown in brackets, were 46.0 [30.0–69.0] s for DM, 109.5 [78.0–150.8] s for DBT with DM, and 50.0 [35.0–71.0] s for SM.

**Table 4:** Mean reading time by imaging modality and reader DBT experience

Reader group	Modality	Mean (sec)	95% CI	Vs. DBT + DM (p)	Vs. DM (p)
All readers (n = 12)	SM	57.45	47.05–67.84	< 0.001	0.232
Experienced readers (n = 5)	SM	55.9	39.56–72.24	< 0.001	0.12
Novice readers (n = 7)	SM	58.55	44.63–72.47	< 0.001	0.801
All readers (n = 12)	DBT + DM	122.58	112.19–132.98	reference	< 0.001
Experienced readers (n = 5)	DBT + DM	121.43	105.09–137.77	reference	< 0.001
Novice readers (n = 7)	DBT + DM	123.41	109.49–137.33	reference	< 0.001
All readers (n = 12)	DM	55.4	45.00–65.79	< 0.001	Reference
Experienced readers (n = 5)	DM	51.77	35.43–68.11	< 0.001	Reference
Novice readers (n = 7)	DM	57.99	44.07–70.90	< 0.001	Reference

CI, confidence interval; SM, synthesized mammography; DBT, digital breast tomosynthesis; DM, digital mammography

*Statistically significant difference (p < 0.05). Reading times represent mean values across readers within each group

## Discussion

In this multireader, multicase study, SM alone showed no statistically significant difference in AUC compared with DBT with DM or DM alone, demonstrated higher sensitivity than DM without compromising specificity, and reduced reading time by 53% and radiation dose by 39%. Inter-reader agreement was substantial across all three modalities, indicating consistent diagnostic performance among readers.

SM alone showed no significant difference in AUC compared with DBT with DM and demonstrated higher cancer detection sensitivity. Among experienced readers, both AUC and sensitivity with SM were significantly greater than with DM, consistent with previous studies [11, 12]. The superior sensitivity of SM over DM supports its potential use as a standalone 2D mammographic modality, which may facilitate broader radiologist adoption. In contrast, this advantage was not observed among novice readers, who showed similar AUC and sensitivity across SM, DBT with DM, and DM. Furthermore, the improvement in AUC among experienced readers, although statistically significant, was modest and should therefore be interpreted cautiously in terms of clinical impact. Nevertheless, experienced readers demonstrated improved diagnostic performance with DBT with DM compared with DM alone, with further improvement observed with SM compared with DBT with DM, representing a key finding of this study. In population-based screening settings, even small improvements in diagnostic performance may translate into meaningful clinical impact at the population level.

While reader experience and case volume affect mammographic performance [18] and DBT has been reported to improve accuracy regardless of experience [19], our findings suggest that a certain level of experience may be required for optimal DBT and SM interpretation. Because the image characteristics of DBT and SM differ from those of DM due to image processing and reconstruction algorithms [20], experience with DM alone may be insufficient. Notably, the additional improvement with SM was confined to experienced readers, suggesting that effective SM interpretation may benefit from prior familiarity with DBT. As experienced readers also lacked prior SM experience, the lower performance among novice readers may reflect unfamiliarity with DBT-related image characteristics rather than an inherent limitation of SM. Because SM shares image characteristics more closely with DBT than with DM, familiarity with DBT may facilitate its interpretation, which could partly explain the improved performance among experienced readers and the limited advantage observed among novice readers. SM preserves key advantages of DBT, including clearer lesion margins and improved visualization of lesion structure, which may be better leveraged by readers trained in DBT interpretation [21]. Collectively, these findings warrant further investigation through targeted training and prospective validation. Given the sensitivity–specificity trade-off, SM achieved the highest sensitivity but lower specificity, although the differences in specificity were not statistically significant. A learning curve for SM likely exists; readers may not be fully proficient despite training. With experience, specificity may rise without sensitivity loss, improving SM performance.

**Fig. 5:**
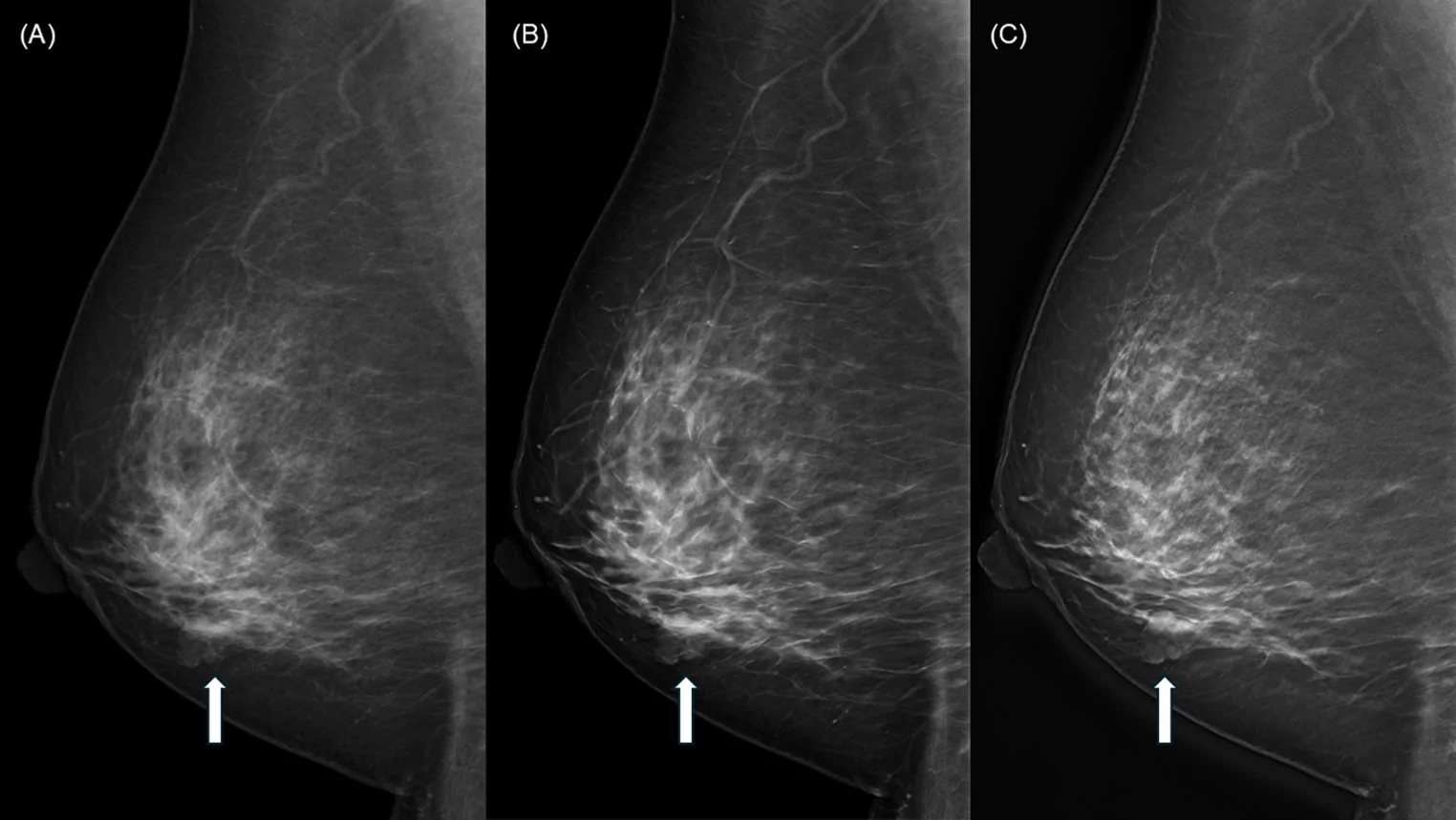
Images from a 67-year-old Japanese woman with invasive ductal carcinoma in a heterogeneously dense right breast. **A** lobulated small mass (arrow) protruding from the inferior margin of the fibroglandular tissue into the subcutaneous fat is more clearly visualized on the digital breast tomosynthesis (DBT) image (**C**) than on the corresponding synthesized mammogram (SM) (**B**) or the digital mammogram (DM) (**A**)

The following subgroup analyses were exploratory in nature and should be interpreted with appropriate caution. In calcification-only lesions, SM showed higher sensitivity among experienced readers. Prior studies suggest potential utility of SM for calcification detection [11, 12, 22], and our results indicate that improved image contrast and reduced noise may contribute to this observation. In addition, differences in spatial resolution and image texture between SM and DM may also influence lesion conspicuity, particularly for fine structures such as microcalcifications and subtle architectural distortions. For non-calcification-only lesions, SM showed higher sensitivity among experienced readers, consistent with prior reports [12]. Our results suggest that the new algorithm may improve image quality for non-calcified lesions, while reduced contrast may still occur in regions with steep density transitions (Fig. 5).

When interpreting SM, readers should be aware that AI-based synthesis and filtering processes may modify lesion appearance; for example, margins of masses or architectural distortions may appear altered, and the visibility of microcalcifications may be affected. Familiarity with DBT-derived image characteristics is therefore important for accurate interpretation.

In dense breasts, SM showed higher sensitivity among experienced readers. While prior meta-analyses, largely in Western populations, have reported improved sensitivity with DBT in dense breasts [23], differences in breast density distribution and assessment, particularly in Asian populations [24, 25], warrant further investigation. In non-dense breasts, SM showed higher sensitivity than DM among experienced readers, with a similar trend compared with DBT with DM. Prior meta-analyses suggest limited additional benefit of DM when DBT is used [26], indicating that SM alone may be sufficient in selected non-dense cases.

This study found that SM reduced reading time by 53.1% compared with DBT with DM, a reduction comparable to that reported for AI-assisted DBT alone [4, 27]. As this study evaluated DBT with DM, SM may serve as a more efficient AI-supported alternative. Time savings were consistent for novice and experienced readers. As long reading time limits DBT adoption [28], SM may help overcome this barrier [11].

In this study, SM derived from DBT delivered a mean dose of 1.73 mGy, 　which is approximately 39% lower than that for DBT with DM. These results, consistent with prior studies [11, 12], further support SM as a viable screening option.

These findings suggest that SM may serve as a standalone modality in many cases, with selective DBT review when clinically indicated, although further local validation is required. This approach may improve reading efficiency and support more sustainable population-based screening. As an AI-supported method derived from DBT data, SM is designed to augment expert interpretation by optimizing image quality without CAD-like diagnostic or reader assistance, potentially mitigating medicolegal and ethical concerns. Broader adoption may be constrained by workflow integration, vendor availability, and regulatory or reimbursement factors; moreover, this study does not support the independent clinical use of SM without access to the acquired DBT images, and DBT should remain available for review when additional diagnostic clarification is required.

Several limitations should be acknowledged. First, only MLO views were used, which may affect sensitivity and specificity because craniocaudal views were not included [9]. This design reflects Japanese screening practice for women aged ≥ 50 years but may limit generalizability to two-view protocols used elsewhere. Second, the cancer-enriched laboratory dataset limits generalizability to population-based screening with low cancer prevalence [29]. This enrichment may also affect reader behavior and diagnostic thresholds compared with real-world screening settings. In addition, tumors larger than 30 mm were excluded, which may limit the applicability of our findings primarily to smaller, screening-detected lesions. Third, the small number of benign lesions precluded reliable lesion-specific specificity analyses. Fourth, inclusion of readers without prior DBT experience may have influenced comparative performance across modalities. Fifth, SM is generated from DBT data through both acquisition-dependent reconstruction processes and AI-based algorithms; therefore, it cannot be considered fully independent from DBT, and the observed differences in diagnostic performance cannot be attributed solely to the imaging modality itself, although this integrated nature reflects real-world clinical imaging workflows. In addition, AI-based processing was applied only to SM and not to DBT images; therefore, the comparison does not reflect fully equivalent AI-enhanced conditions across modalities, which may have influenced the observed differences. Finally, the AI algorithm requires further validation in multicenter studies with more diverse populations. Despite these limitations, the study was designed to simulate a screening setting, with recall-based assessments performed without prior case information. To our knowledge, this is the first adequately powered study comparing AI-supported SM, DBT with DM, and DM alone under screening-like conditions.

In conclusion, in this retrospective multireader study using a mixed dataset of screening recalls and symptomatic cases, SM derived from DBT showed diagnostic performance comparable to DBT with DM or DM alone, while reducing reading time and radiation dose. These findings should be interpreted cautiously and are intended to inform screening-oriented reading workflows rather than to support stand-alone screening use. Further prospective validation in population-based screening cohorts is required to clarify the role of SM and the optimal use of DBT as an adjunct.

## Abbreviations

AI: Artificial intelligence
SM: Synthesized mammography
DBT: Digital breast tomosynthesis
DM: Digital mammography
MLO: Mediolateral oblique
CAD: Computer-aided detection
AUC: Area under the receiver operating characteristic curve
POM: Probability of malignancy
